# Technical Optimization of Decompressive Craniectomy for Possible Conversion to Hinge Craniotomy in Traumatic Brain Injury

**DOI:** 10.7759/cureus.39767

**Published:** 2023-05-31

**Authors:** Abdul-Kareem Ahmed, Pemla Jagtiani, Salazar Jones

**Affiliations:** 1 Neurosurgery, University of Maryland School of Medicine, Baltimore, USA; 2 Medical School, State University of New York Downstate Health Sciences University, New York, USA; 3 Neurological Surgery, Mount Sinai Hospital, New York, USA

**Keywords:** bone flap, elevated intracranial pressure, traumatic brain injury, hinge craniotomy, decompressive craniectomy

## Abstract

Hinge craniotomy for the management of elevated intracranial pressure (ICP) in traumatic brain injury remains a technique not widely adopted. The hinged bone flap decreases the allowable intracranial volume expansion, which can lead to persistent post-operative elevated ICP and the need for salvage craniectomy. Herein, we describe the technical nuances in performing a decompressive craniectomy that, when optimized, allows for stronger consideration for hinge craniotomy as a definitive technique. To conclude, hinge craniotomy is a reasonable option in the setting of traumatic brain injury. Trauma neurosurgeons can consider the technical steps to optimize a decompressive craniectomy and perform hinge craniotomy when allowable.

## Introduction

Decompressive craniectomy (DC) for refractory elevated intracranial pressure (ICP) is a life-saving surgical procedure with proven benefits in the traumatic brain injury (TBI) population [1]. Decompressive craniectomies, however, are fraught with hemorrhagic, infectious, and cerebrospinal fluid complications which have been extensively reviewed [2].

Hinge craniotomy (HC) has been described as an alternative to DC as it allows for ICP relief and negates the need for a cranioplasty [3]. The offered advantages of ICP control without a need for staged cranioplasty may offer better resource utilization and lower healthcare costs with similar outcomes [4-6]. HC is not widely utilized, and the literature still consists mainly of single-center experiences [3,5,7]. HC has found a role more commonly in low- and middle-income countries [8]. There are several criticisms of the HC including lack of decompression over the hemisphere proper, particularly along the hinge. The retained bone flap decreases the allowable space for an edematous brain to occupy, potentially resulting in the need for a salvage DC in a critical patient. In a recent scoping review, nine of 283 cases of HC required salvage DC (3.2%) [7].

While the criticisms of HC are valid, the review suggests comparable results between HC and DC in terms of ICP control, mortality, and functional outcomes [7]. Certainly, more space is available to accommodate brain swelling with DC, however, extensive extracranial brain herniation is not always required to relieve elevated intracranial pressure. Multiple studies have shown that ICP is already decreased below 20 mmHg with bone flap removal, with additional ICP reduction at the time of durotomy [9-11]. Additionally, in-vitro and radiographic studies have demonstrated that excessive mushrooming of the brain through craniectomy defects causes axonal stretch resulting in further injury, a delayed response in returning to baseline length, and resultant neurodegeneration [12-14].

In this paper, we examine the technique of performing a DC with a focus on steps that may potentially undermine the benefit of the procedure. We put forward that meticulous surgical technique will not only maximize decompression but allow for the consideration and success of HC.

The technique of DC has been described by multiple authors. Descriptions and variations among techniques focus on the scalp incision, creation of the bone flap, and dural opening. While these steps are certainly important, there are several other measures that require thought and attention to maximize the benefit of the DC. Without these considerations, the surgeon may encounter pitfalls that can minimize the extent of decompression or even worsen compression. By avoiding the technical pitfalls of a DC, decompression can be optimized, which can allow the consideration of converting to an HC.

## Technical report

Temporalis muscle elevation

**Figure 1:**
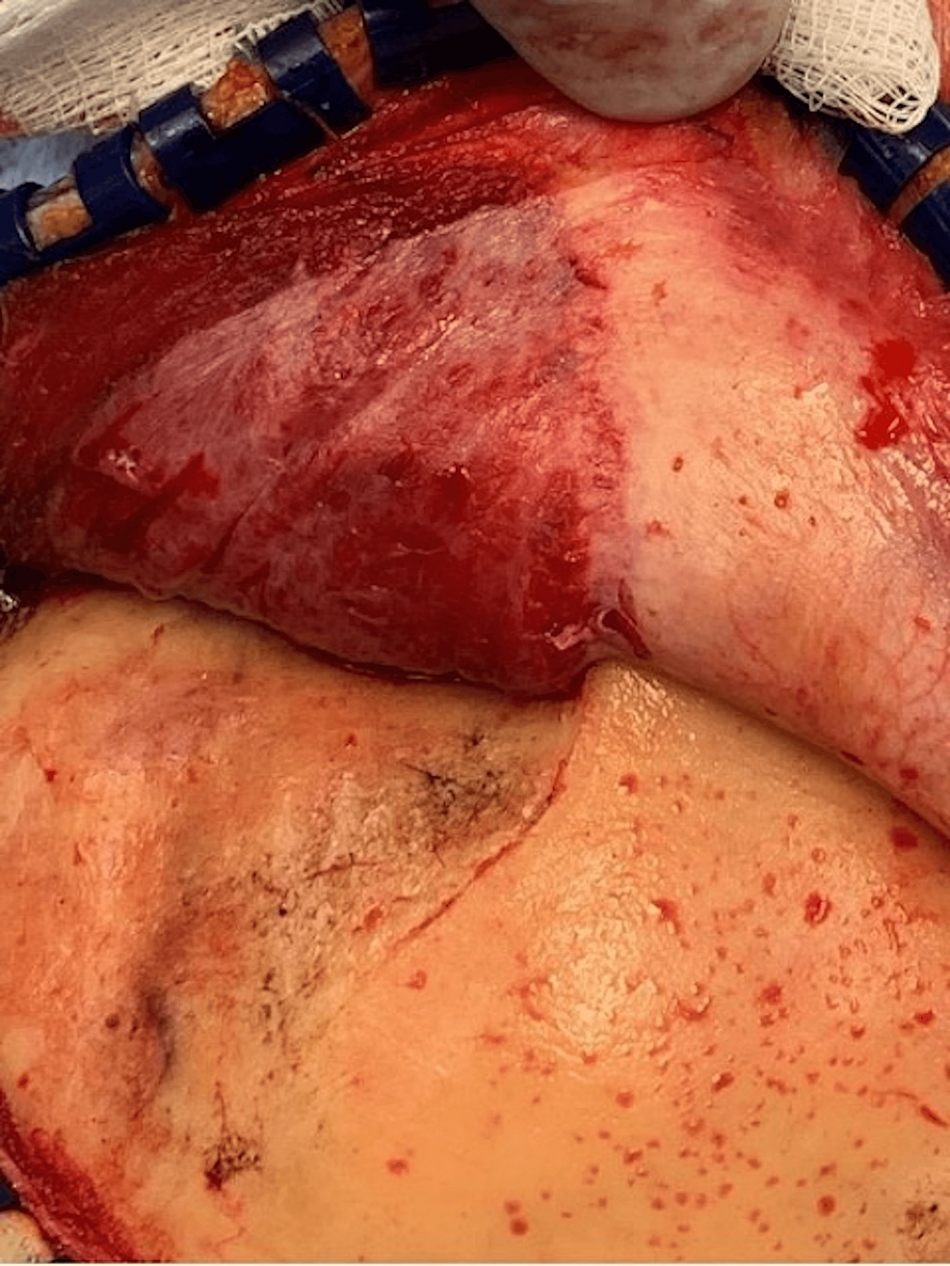
Preservation of the pericranium keeps the temporalis elongated and atraumatic. Upon scalp closure, the insertion of the temporalis will rest over the superior temporal line rather than contract over the middle fossa. Image courtesy Dr. Salazar Jones.

**Figure 2:**
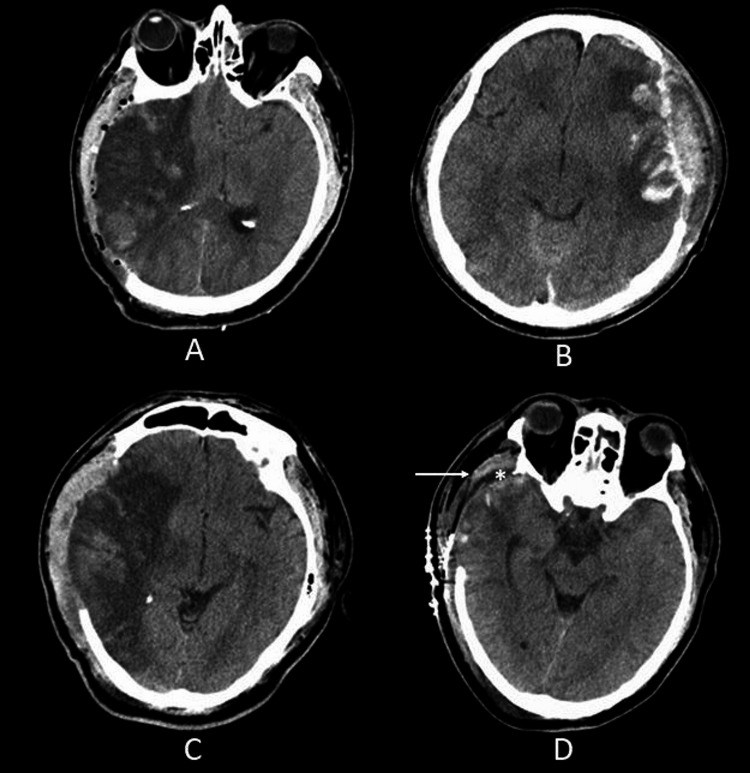
Representative axial computed tomography (CT) slices showing: swollen right temporalis muscle (A); left temporalis hematoma with indentation of the dura, brain compression, and midline shift (B); swollen right temporalis muscle and subgaleal hematoma (C); preserved right temporalis muscle (arrow) with additional space (asterisk) over the temporal lobe occupied by CSF (D). Image courtesy Dr. Salazar Jones.

Shortly after the incision is made, the temporalis muscle must be properly elevated with preservation of the pericranium and attachment along the scalp, as described by Kadri and Al-Mefty [15]. Preservation of the pericranium minimizes trauma to the temporalis muscle and contraction of its fibers (see Figure 1). When the temporalis fibers contract, the resulting muscle mass becomes centered over the middle fossa floor. If the pericranium is destroyed, there is increased trauma to the temporalis muscle that leads to swelling of the muscle, intramuscular hematomas, and epidural hematomas (Figure 2). The juxtaposition near the temporal lobe can create a mass lesion that counters the goals of the decompressive craniectomy. Other authors have suggested temporalis muscle resection to address temporalis complications. However, resection of the temporalis leads to deficits with mastication and poor cosmesis. It is preferable to respect the temporalis muscle to prevent temporalis complications rather than to resect normal functional tissue. 

Craniotomy

**Figure 3:**
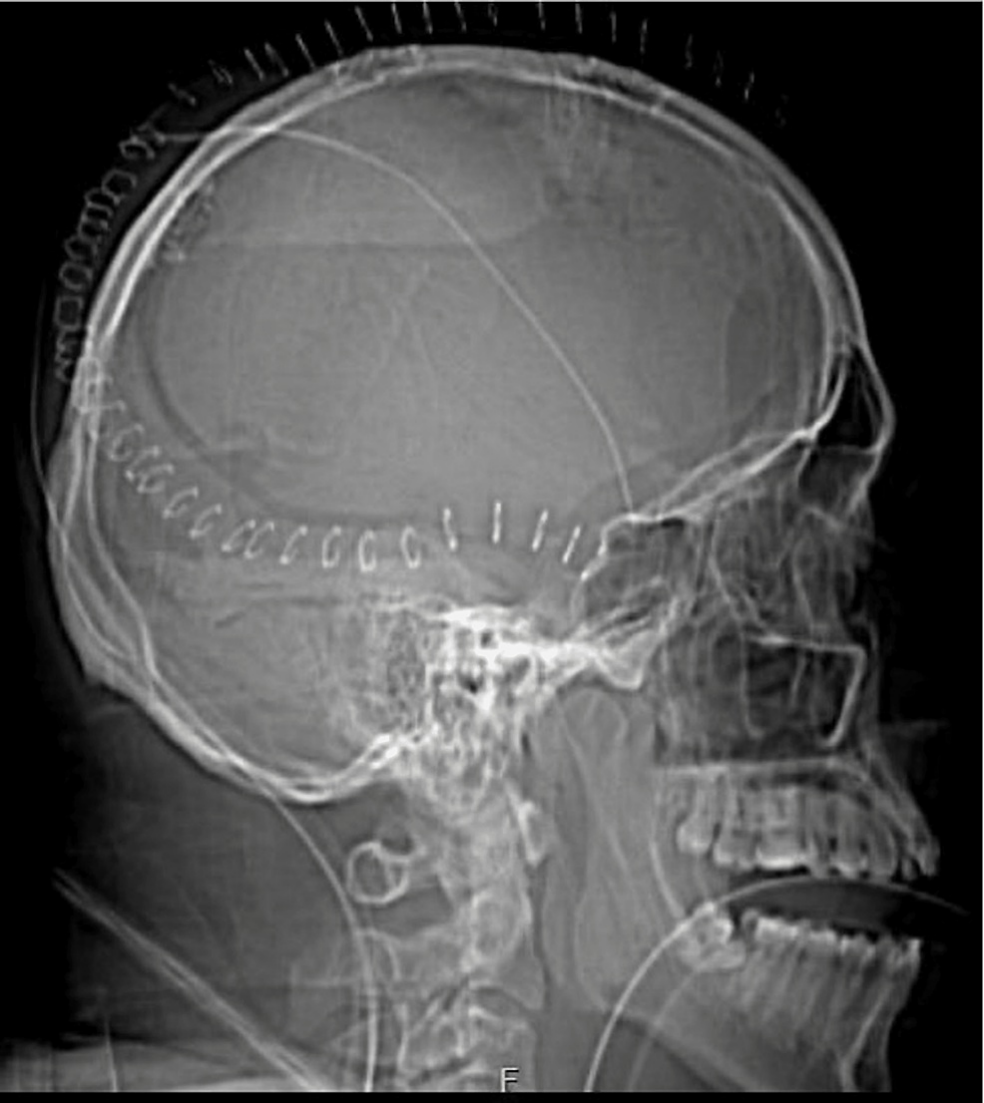
Lateral CT scout image showing large craniotomy bone flap with cranial fixation plates along the medial aspect to allow for the hinge. Image courtesy Dr. Salazar Jones.

Proper handling of the temporalis muscle allows for easy recognition of the posterior root of the zygoma and middle fossa floor. A bone flap size of 12 cm x 15 cm is recommended (Figure 3) [16]. After performing a large craniotomy, the residual squamous temporal bone should be removed down to the middle fossa floor. The subtemporal decompression is thoughtful even in instances where the bone will be replaced. An inadequate bone flap will result in compression of the brain and cortical vessels at the bone edge.

Dural opening

**Figure 4:**
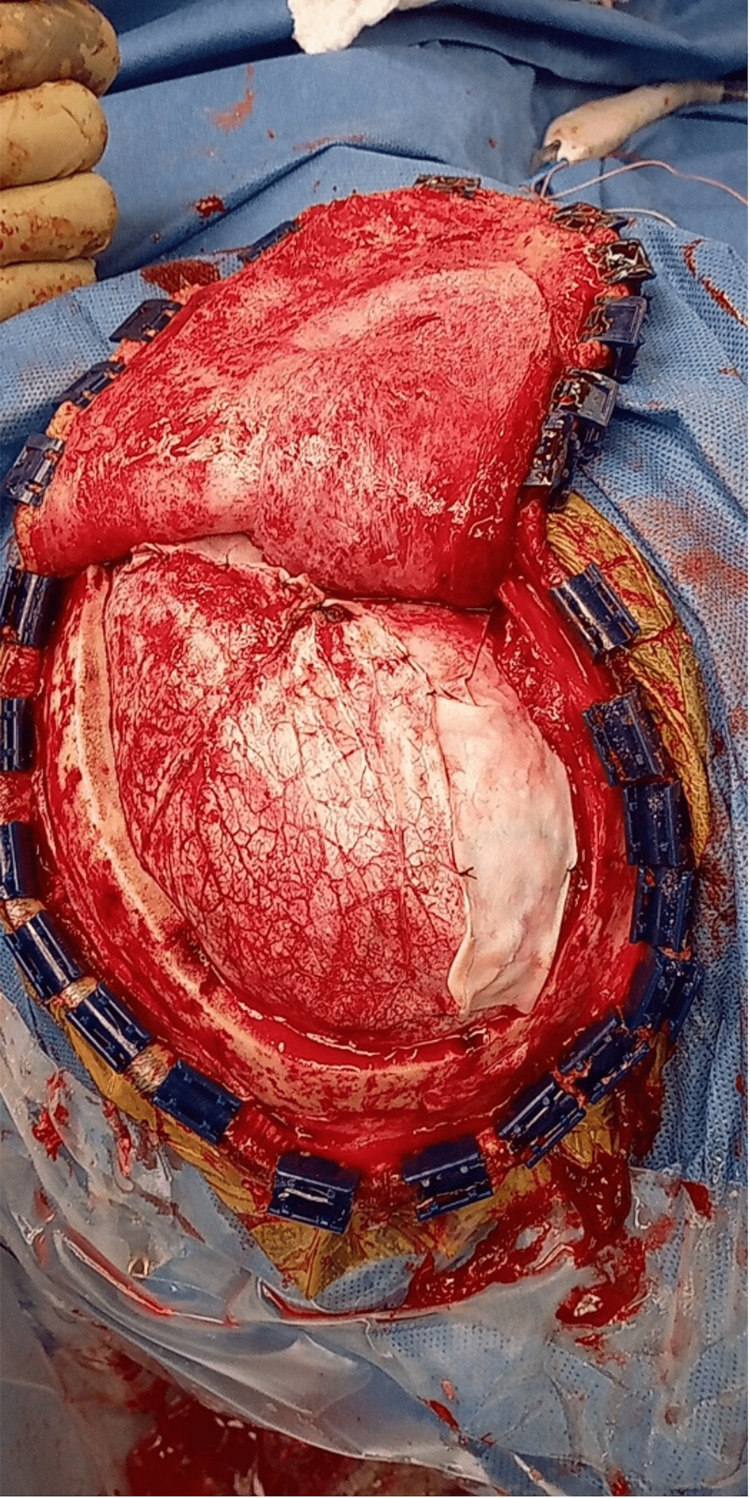
Intraoperative depiction after large right-sided craniotomy and expansile duraplasty. Image courtesy Dr. Salazar Jones.

There are many descriptions of dural openings for decompressive craniectomies. Intracranial pressure is significantly reduced at two steps of the surgery: bone flap removal and dural opening [9-11]. Rapid opening of the dura leads to extensive intraoperative brain swelling [17]. This “mushrooming” of the brain is not a true edema. The pathophysiology is not fully understood but is thought to involve hyperemia and vascular engorgement [11]. Extensive brain herniation will lead to compression of cortical vessels along the bone edge akin to an undersized bone flap. Variations of controlled dural opening with discontinuous cuts or slits have been described [1]. A controlled opening appears to allow for the relief of elevated ICP while reducing intraoperative brain swelling [11,17]. There is some data to suggest a controlled dural opening results in improved outcomes [11]. In our experience, a tense brain prior to dural opening will become soft with a controlled dural opening. This observation supports the likely involvement of vascular engorgement with a rapid dural opening. The dura is then further opened for performing an expansile duraplasty (Figure 4).

Resection of frontal or temporal contusion

In instances where the bone flap is replaced, the progression of traumatic hemorrhagic contusions is a reason for reoperation or salvage decompressive craniectomy [18]. Thus, proper recognition of contusions should be done by preoperative CT and intraoperative inspection. Surgical evacuation of frontal or temporal contusions greater than 20 cm^3^ has been recommended by the Surgical Management of TBI Author Group [19]. For significant hemorrhagic temporal contusions, subpial resection of the temporal contusion can be performed. Resection typically reveals necrotic brain and blood products. The resection bed can be a site for post-operative hemorrhage, so it must be done with attention to proper hemostasis.

## Discussion

To hinge or not?

**Figure 5:**
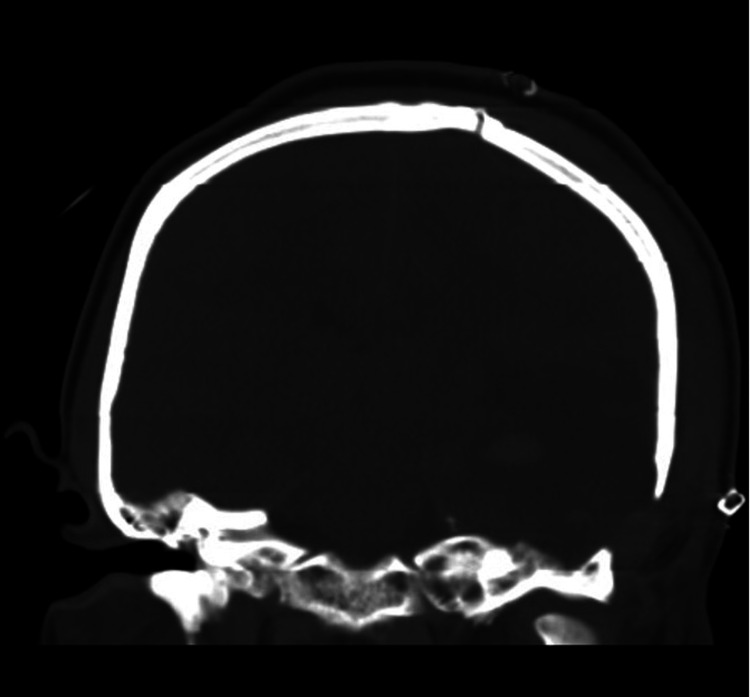
Coronal CT bone window showing the expansion of the bone flap with the use of the hinge. Image courtesy Dr. Salazar Jones.

**Figure 6:**
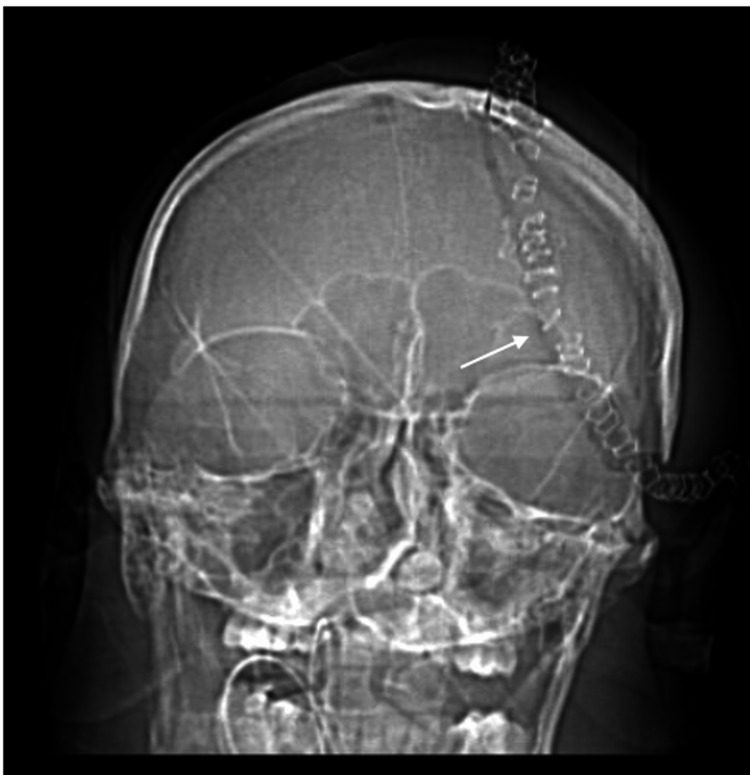
AP CT scout image showing that the craniotomy flap has hinged outward. Image courtesy Dr. Salazar Jones.

As the expansile duraplasty is being finished, the decision on whether to replace the bone flap and perform an HC must be made. The decision begins with a proper assessment of the brain for adequate decompression and potential for further swelling. Attention should be paid to the extent of brain contusions, color, pulsatility, and turgidity of the brain. We will replace the bone flap if it comfortably rests in an anatomic or near anatomic position with a hinge along the midline for cranial expansion if needed (Figures 5-6).

If the brain appears too contused, is not pulsatile, or is tense, leaving the bone off is recommended, which provides maximal decompression. The additional morbidity from the craniectomy and subsequent cranioplasty would necessitate the patient survives the acute period, which may not be likely. Additionally, direct trauma or fractures that result in a preoperative swollen temporalis can impact the ability of the bone flap to hinge. Sometimes a hematoma within the temporalis can be evacuated, but the swelling often still ensues.

Significant frontal and temporal contusions on the dominant hemisphere may require evacuating the mass lesions, however, it is recommended against performing any significant combined frontal and temporal lobectomy on the dominant hemisphere. 

Hinge craniotomy has been reported in the settings of large MCA infarcts [3,5,8]. Unlike TBI where there are heterogeneous, albeit, diffuse, areas with a different propensity to swell, a large MCA infarct consists of a vast expanse that may all swell. The hinge craniotomy preferentially decompresses the brain along the skull base furthest from the hinge point. This is congruent with the common sites of traumatic contusions being in the frontal and temporal lobes. Expansion of intracranial volume can sufficiently occur along the hinge without a detrimental rise in ICP [20]. Malignant cerebral edema from large MCA infarcts involves higher areas closer to the hinge point. We recommend reservation in performing a hinge craniotomy for this entity especially if an “early” decompressive craniectomy is being performed for an MCA infarct as the extent of future swelling is not known.

## Conclusions

DC remains a workhorse technique for neurosurgeons. We described critical surgical steps to relieve elevated ICPs while reducing untoward surgical complications. These steps should be considered for all decompressive cases. A small craniotomy, severe intraoperative brain swelling, and temporalis swelling/hematomas only negatively impact surgical outcome. When performing a craniectomy, it may not be necessary to evacuate a hemorrhagic contusion, however, the limited data and recommendation suggest a benefit to doing so, irrespective of intent to replace the bone flap. HC should not be indiscriminately used and expected to work. The pitfalls that negatively impact a DC are only amplified with a HC. The final decision to perform a HC should be reserved until after the decompression is performed with prior steps optimizing the scenario. With adequate decompression and consideration of pitfalls, HC can be an attractive option for TBI requiring surgical control of ICP.
